# 

**Published:** 1898-08

**Authors:** 


Vol. LII. AUGUST, 1898. No. 8.

LIBRARY
SURGEON GENERAL'S OFFICE
AUG.-15.-1898

### THE DENTAL REGISTER

A MONTHLY JOURNAL.

Devoted to the Interests of the Profession.

---

EDITED BY
J. TAFT, D. D. S.

---

PUBLISHED BY
SAM'L A. CROCKER & CO.,
OHIO DENTAL AND SURGICAL DEPOT,
35, 37 and 39 West Fifth Street, CINCINNATI, OHIO.

Entered at the Postoffice at Cincinnati, O., as second class mail matter.

TERMS: $2.00 PER ANNUM IN ADVANCE. SINGLE COPIES, 25 CTS.

